# Understanding the contribution of toxicant exposures to Alzheimer’s disease and related dementias

**DOI:** 10.1016/j.conb.2026.103192

**Published:** 2026-04-09

**Authors:** Gareth R. Howell, Paul R. Territo, David Aylor, Lee E. Goldstein

**Affiliations:** 1 The Jackson Laboratory, Bar Harbor, ME, 04609, USA; 2 Graduate School of Biomedical Sciences, Tufts University, Boston, MA, USA; 3 Graduate School of Biomedical Sciences and Engineering, University of Maine, Orono, USA; 4 Stark Neurosciences Research Institute, Indiana University School of Medicine, Indianapolis, IN, 46202, USA; 5 Department of Medicine, Division of Clinical Pharmacology, Indiana University School of Medicine, Indianapolis, IN, 46202, USA; 6 Center for Human Health and the Environment, Bioinformatics Research Center, Department of Biological Sciences, North Carolina State University, Raleigh, NC, 27695, USA; 7 Boston University Alzheimer’s Disease and CTE Center, Boston University School of Medicine, Boston, MA, USA; 8 Department of Neurology, Boston University School of Medicine, Boston, MA, USA; 9 Department of Pathology, Boston University School of Medicine, Boston, MA, USA; 10 Department of Psychiatry, Boston University School of Medicine, Boston, MA, USA; 11 Department of Ophthalmology, Boston University School of Medicine, Boston, MA, USA; 12 Department of Biomedical Engineering, Boston University College of Engineering, Boston, MA, USA; 13 Department of Electrical and Computer Engineering, Boston University College of Engineering, Boston, MA, USA

## Abstract

Alzheimer’s disease and related dementias (AD/ADRD) are modulated by gene-environment (GxE) interactions across the lifespan. Variants of specific genes increase AD risk and synergize with exposures to environmental toxicants (“exposome”), including neurotoxic metals and metalloids such as lead (Pb), cadmium (Cd), and arsenic (As). These neurotoxicants enter the body (via drinking water, contaminated food, and airborne particulates), transit in blood, cross the blood–brain barrier, and distribute in brain where the retained toxicant disrupts central nervous system development, structure, and function. Chronic exposure to these ubiquitous toxicants is common in disadvantaged communities, raising concerns about health risk disparities linked to geographic, socioeconomic, and racial demographics. While Pb, Cd, and As are established human neurotoxicants with suspected linkage to AD/ADRDs, the mechanisms underpinning AD/ADRD-related GxE interactions specific to metal-metalloid toxicant exposures are largely unknown and potentially modifiable. Preclinical models and resources are needed to facilitate research into the underlying mechanisms by which AD genetics and exposome affect brain health, aging, and AD/ADRD pathobiology.

## Introduction

Alzheimer’s disease (AD) risk and health outcomes result from dynamic interaction between genes and environment (GxE interaction) across the lifespan [1–3]. While individual genetics are not readily modifiable, extrinsic factors often are, but only if known, understood, and corrected. Numerous large epidemiological studies (Environmental Influences on Child Health Outcomes, ECHO; Adolescent Brain Cognitive Development, ABCD; All-of-Us, AoU; HEALthy Brain & Child Development, HBCD; Comparative Toxico-genomic DB, CTD; NeurotoxKB) strongly support linkage between exposure to specific environmental toxicants and long-term deleterious effects on neurodevelopment, brain health, cardiovascular/cerebrovascular disease, and age-related neurodegenerative disorders. However, some epidemiological studies failed to show strong association between certain toxicants that may be due to a variety of factors including population specificity, cohort size, or tissue source (e.g., postmortem tissue) [4–6]. Toxicant exposure is common in disadvantaged groups, raising a concern about health disparities and environmental justice [7]. This is likely due to structural and social determinants of health that persist over a lifetime and include economic stability, social and community context, access to and quality of health care, educational/occupational settings, and community/neighborhood-built environments [8]. For instance, low-income housing is often located near current or previously used industrial sites that can increase exposure to toxicants.

The impact of exposure may vary depending on whether the exposure is acute or chronic or the stage(s) of life when exposure occurs. For instance, exposure may happen *in utero* or in early life when the brain is developing or maturing. Alternatively, or in addition, chronic exposure may occur during adulthood such as during a person’s working life, or even acutely at distinct stages such as in midlife or old age. We might speculate exposure during brain development/maturity or chronic exposure during adulthood through midlife as having potentially the greatest impact on Alzheimer’s disease and related dementias (AD/ADRD) risk. However, while generally exposure to toxic metals/metalloids are widespread [7], the impact of acute or chronic exposure during different life stages are among the least-studied AD/ADRD risk factors.

The Agency for Toxic Substances and Disease Registry (ATSDR) Substance Priority List (SPL, Table 1) ranks hazardous substances found at Superfund sites (NPL) by combining frequency of occurrence, toxicity, and potential for human exposure, guiding the agency’s toxicological profiles [7]. Toxicants are scored based on three main factors: *frequency of occurrence*, how often they appear at NPL sites; *toxicity*, their harmful effects on human health; and *potential for human exposure*, and the likelihood and extent of human exposure. Priority is not simply based on human toxicity *per se* but rather a ranking based on potential toxic impact on human populations. In the following, we review the top two hazardous substances (arsenic, As; lead, Pb). In addition, we review cadmium (Cd) as along with lead it is notable for its strong neurotoxic effect even at low levels of exposure [4,6].

**Figure 1:**
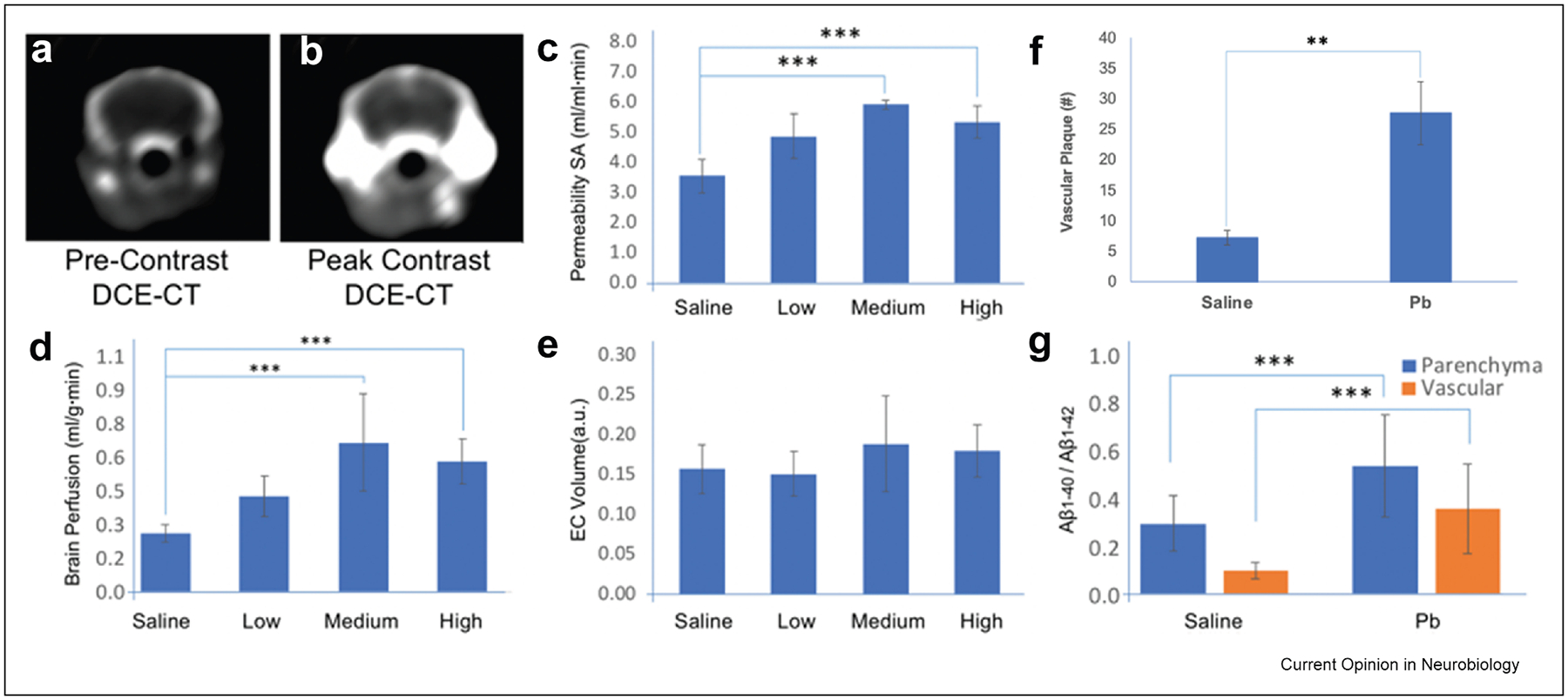
Pb impairs BBB integrity and increases vascular plaque-associated Aβ1–40/Aβ1–42 ratio in B6.APPSwDI mouse brain. *In vivo* DCE-CT: (**a**) precontrast, (**b**) peak contrast. Lead acetate (13.5, 27, 56 mg/kg) vs saline x 1 wk: (**c**), permeability surface area product; (**d,**) tissue perfusion; (**e**), extracellular volume fraction. (**f**), Vascular plaques. (**g**), Aβ1–40/Aβ1–42 ratio (mean ± SEM); ***, p < 0.05, one-tail ANOVA. a–e: Adapted from: Gu et al., 2020; f–g: Adapted from Gu et al., 2024. ANOVA,; **BBB**, blood–brain barrier; DCE-CT, dynamic contrast enhanced-computed tomography; Permeability SA, permeability surface area.

• **Arsenic** (As) is a potent environmental toxicant ranked first on the ATSDR Toxic Substance Priority list [7] and impacts the health of >200M people worldwide [9]. Chronic As exposure affects skin, kidney, heart, and brain [10–13]. The primary exposure route for inorganic As is ingestion of contaminated drinking water and food. While As in drinking water is tightly regulated in the United States (US threshold: 10 μg/L) [14], many Americans are chronically exposed to high As levels via unregulated well water [15]. As-induced phenotypes in mice recapitulate human As toxicity in humans [16–21]. A large literature indicates that As exposure negatively impacts brain development, structure, and function and increases risk for AD/ADRD [22–24]. In mouse models, chronic As exposure increases AD-relevant phenotypes [25]. Cell-based studies corroborate As-induced Aβ and tau proteinopathies and LOAD-related molecular abnormalities [26–30]. Mechanistically, As exposure may increase oxidative stress, chronic neuroinflammation, and mitochondrial dysfunction, all of which have been proposed as causative factors in AD/ADRD pathology [31].

• **Lead** (Pb) is an abundant environmental toxicant that impacts morbidity and mortality globally [7]. Pb ranks second on the ATSDR Toxic Substance Priority List [7] and accounts for ~1% of the global burden of disease (World Health Authority) [32]. Bone contains 70—95% Pb body burden and serves as a releasable Pb reservoir (cumulative toxicant). Even low level Pb (<5 μg/dL) causes irreversible brain damage in children [33,34] and increases all-cause mortality [35]. World Health Authority-Center for Disease Control (WHO-CDC) declared no safe Pb level [36]. In the US, ~400,000 deaths are attributable to Pb each year [35]. Pb is linked to cognitive deficits, dementia, and neurodegenerative diseases (AD, ALS, and PD) in humans and animal models [4,37–52]. Mechanistically, Pb exposure may exacerbate AD/ADRD pathologies through a variety of processes. For instance, transgenic AD mice exposed to Pb show brain biometal (Cu, Zn, and Fe) dyshomeostasis [53]. In two studies, authors showed Pb exposure decreased blood—brain barrier integrity, increased the amount of parenchymal and vascular-associated plaques, and increased Aβ1–40/Aβ1–42 ratios that is considered a feature of cerebral amyloid angiopathy (Figure 1) [54,55].

• **Cadmium** (Cd) is widely used in electric batteries, paints, pigments, industrial coatings, brake linings, electroplated products. Cd ranks seventh on the ATSDR Toxic Substance Priority list, has no known beneficial physiologic function, and is toxic to multiple tissues in humans. Clinical studies link Cd exposure to cognition deficits in older adults [56,57]. NHANES 1999—2004 epidemiology study linked Cd exposure to increased AD risk [58,59]. Cd exposure is linked to renal and cardiovascular disease, osteoporosis, hypertension, diabetes, and neurotoxicity. Cd exposure results from the following: (i) ingestion of contaminated drinking water, food and (ii) inhalation of tobacco smoke, industrial fumes, and polluted air [60]. Cd induces Aβ amyloidopathy, tauopathy, and cognitive deficits in animal models [61,62]. Mechanistically, Cd exposure has been shown to increase oxidative stress, Ca^++^ dyshomeostasis, and neuroinflammation that may cause or synergize with AD/ADRD pathologies, such as amyloid plaques and tau tangles, to increase neuronal cell dysfunction and neurodegeneration [63–65].

**Figure 2:**
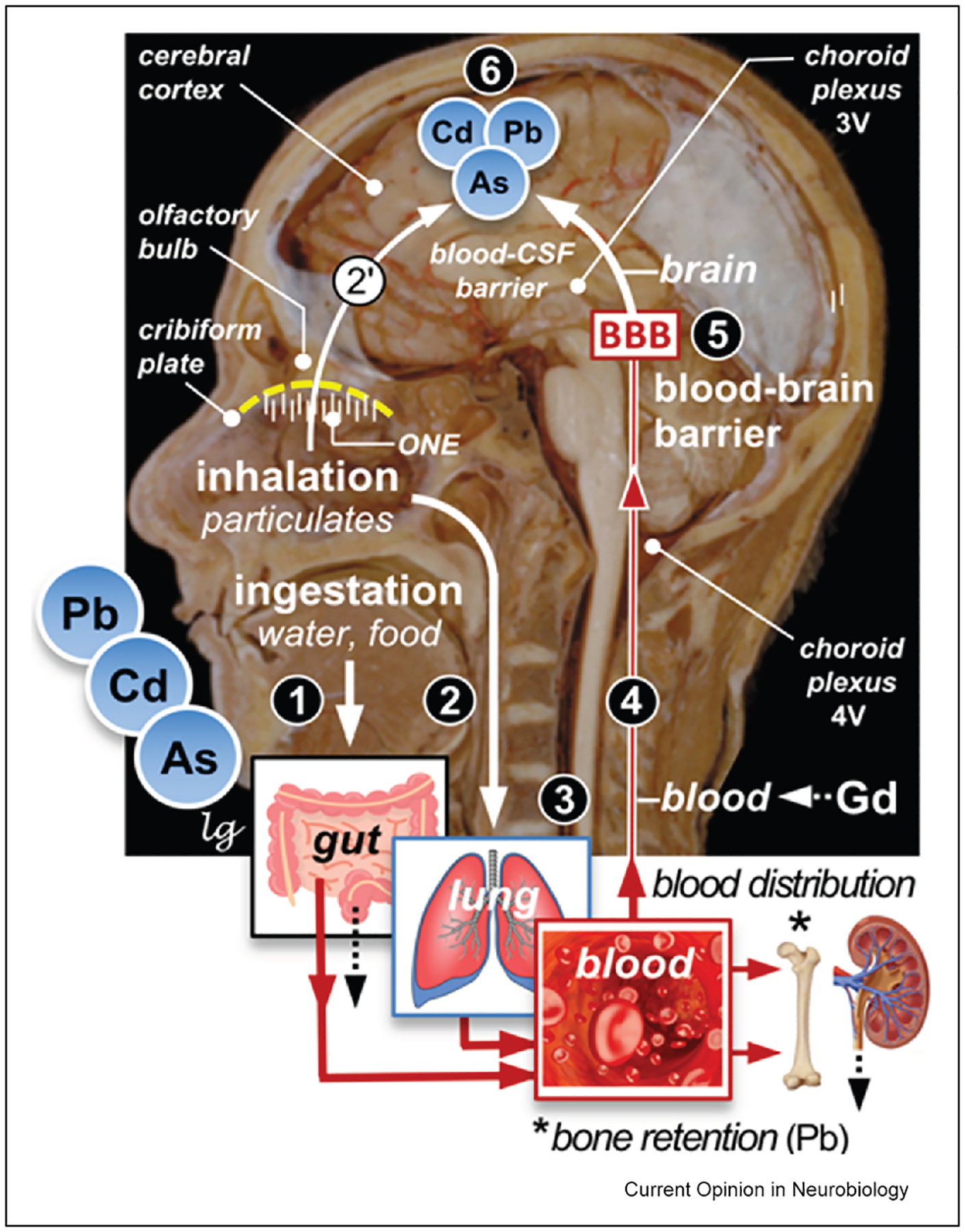
Pb, Cd, and As neurotoxicants’ exposures, bodily distribution, and transport to brain. 1° route: ingestion (1), contaminated water, food. 2° route: inhalation (2), particulates. Transport via blood (3,4), brain uptake (5,6; via blood–brain barrier, BBB; blood-CSF barrier, BCB). Toxicants localize/persist in discrete regions of brain (toxicant-specific long-term retention). Minor olfactory uptake pathway (Cdand As). Gd exposure is exclusively iatrogenic (Gd-based MRI contrast, i.v.). Pb, Cd, and As are potent neurotoxins that harm brain development, structure, and function. All three modulate canonical AD pathways. However, the mechanisms underpinning Pb, Cd, and As uptake, distribution, retention, toxicity, and AD-linked dependencies (sex, age, genotype, neurodevelopment, Fe status, etc.) are poorly understood. Courtesy of Lee Goldstein, M.D., PhD., Boston University School of Medicine. AD, Alzheimer’s disease; MRI, magnetic resonance imaging.

Pb, Cd, and As enter the body (commonly via contaminated drinking water, food, or airborne particles) and transit to brain via blood where these toxicants disrupt neuronal function and can modulate canonical AD/ADRD-relevant pathways (Figure 2, and reviewed in Ref. [4]). However, more work is needed to determine how combinations of AD/ADRD genetic risk factors interact with exposure-related mechanisms to drive increased risk for AD/ADRD.

## Improving mouse models to test gene by toxicant interactions

Current research offers many instructive examples of relevant associations between environmental exposures and AD/ADRDs, especially in people with genetic susceptibility (e.g. *APOE4*) [3,66,67]. While evidence for AD/ADRD-exposome associations is known, the pathways and mechanisms underpinning linkage are not well understood. Many studies have used animal (including rodent and nonhuman primate) models, and cell models (e.g. Refs. [68,69]) to improve our understanding of how toxicant exposures modify brain health. However, conceptual models and preclinical research aimed at elucidating causal mechanisms contributing to AD/ADRDs have been slow to incorporate new information about the exposome and new model systems for mechanistic investigation of gene by toxicant interactions and latent effects of toxicant exposures (i.e. early, midlife, chronic) [3].

New genetic models should now be considered to enhance the translatability of exposome research. Since 2016, MODEL-AD (Model Organism Development and Evaluation for Late-Onset AD), supported by the National Institute on Aging, NIH, has created more than 70 human-relevant LOAD mouse models with 33 different combinations of human risk and susceptibility alleles. The IU/JAX/PITT MODEL-AD Center has created 3 platform strains (LOAD1, LOAD2, and LOAD3) carrying combinations of humanized *APP* (hAβ), *MAPT* (hTau), and genetic risk factors (APOE-ε4, Trem2*R47H). The *APOE-ε4* strain develops neuro-vascular metabolic uncoupling in a sex-dependent manner. LOAD1 mice express humanized *APOE-ε4* and Trem2*R47H [70]. LOAD2 added hAβ sequence to increase LOAD-relevant Aβ toxicity and amyloidopathy. The importance of GxE exposome interactions in LOAD pathobiology was shown. LOAD2 mice fed high-fat/high-sugar diet (HFD, from 2 months of age) were evaluated at 12 and 18 months of age [71]. LOAD2 mice fed a HFD accumulated insoluble Aβ, showed subtle but significant neuron loss, metabolic/vascular uncoupling, proteomic profiles consistent with synapse loss and cognitive deficits consistent with human LOAD [71]. Recently, the IU/JAX/PITT MODEL-AD Center has created the LOAD3 platform mouse strain that is triple homozygous for humanized *APOE4* (ε4,ε4), humanized Aβ (hAβ), and humanized *MAPT* (hTau) and expresses 3R, 4R hTau isoforms. These mice, which do not develop hallmark pathologies of AD such as amyloid and tau pathology, are ideal to test human-relevant interactions between genetic risk and toxicant exposure. For instance, genetic factors and toxicant exposures may synergize to induce amyloid or tau pathology or perturb canoncical systems, such as cerebrovascular deficits, independent of hallmark pathologies.

The IU/JAX/PITT MODEL-AD Center has also pioneered innovative strategies to align and match mouse models to human LOAD [72]. Generation of human multiomic data (transcriptomics, proteomics, and metabolomics) from human AD cohorts (NIA AMP-AD) has identified LOAD-relevant molecular signatures (e.g. Ref. [73]). These data enable systematic murine-to-human alignment of genes, proteins, and pathways for classification of genetic variation, environmental perturbations, and gene by environment interactions into AD-relevant physiological modules [70,72]. MODEL-AD deploys this strategy to prioritize LOAD mouse models for deep phenotyping and preclinical testing.

## Tracking toxicant exposure through epigenetic modification

An unmet need is to identify those exposed to particular toxicants and to predict the long-term effects of toxicant exposure on AD/ADRD risk. Many chemical toxicants are measurable during or immediately after exposure but epigenetic modifications associated with exposure may modify cells in a manner that is persistent long afterward. Epigenetic profiles measure chemical modifications to DNA and DNA-associated proteins and there is ample evidence that toxicant exposures change these profiles [74,75]. This opens the possibility of developing epigenetic biomarkers of past chemical exposures. Ideal biomarkers of exposure will be toxicant-specific, persistent for months or years in humans, and unaltered by individual variation (e.g. genetics, sex, age). The NIEHS-funded Toxicant Exposures and Responses by Genomic and Epigenomic Regulators of Transcription (TaRGET II) consortium has worked on mouse models to identify the epigenetic patterns associated with a wide range of toxicant exposures, including Pb and As [76]. TaRGET II can serve as a model for AD/ADRD, which can potentially extend those results to incorporate not only epigenetic markers of exposure but also epigenetic markers of disease and disease progression. Elucidating epigenetic mechanisms for environmentally induced disease remains a challenge in the field but has shown promise for some classes of toxicants (reviewed in Ref. [77]).

Highlighting the potential of tracking exposure risk in ADRDs through epigenetics, studies have shown an association among epigenetic effects of environmental exposures in AD populations [78,79]. Mechanistically, it will be important to determine specific epigenetic marks that can be tracked, as well the specific cell types that might be affected. Much of the genetics of AD points toward myeloid cells as drivers of AD/ADRD risk, and so one might speculate particular attention might be paid to blood cells of the myeloid cell lineage.

## Conclusion and future considerations

Toxicant exposure is predicted to make a significant contribution to risk of AD/ADRD and yet our knowledge of the mechanisms underlying this increased risk is still to be fully elucidated. To tackle this, the National Institute on Aging, in collaboration with other institutes within the National Institutes of Health, recently established multiple cross-cutting *TOX-AD consortia* taking innovative and synergistic approaches in humans, mouse models, and cells, to generate much needed resources for the community. The consortium includes three preclinical TOX-AD centers focused on using mouse models (e.g. LOAD3, AD-BXD [80]) to understand the contribution of toxicant exposure (including As, Pb, Cd, wildfire and microplastics) to AD risk. Centers are located at the University of New Mexico, University of Michigan, and our own located across Boston University, Indiana University, and The Jackson Laboratory. The accessibility of these data, tools, and resources to the larger scientific community will accelerate the identification of mechanisms driving toxicant-related damage, improve tracking of those acutely and chronically exposed to dangerous toxicants, and ultimately offer new hope for therapies for AD/ADRD.

## Figures and Tables

**Table 1:** Top 10 ATSDR Substance Priority List [7].

2022 Rank	Substance name	Total points	CAS RN
1	Arsenic	1675	7440-38-2
2	^a^Lead	1531	7439-92-1
3	^a^Mercury	1455	7439-97-6
4	^a^Vinyl chloride	1355	75-01-4
5	Polychlorinated biphenyls	1342	1336-36-3
6	Benzene	1328	71-43-2
7	Cadmium	1317	7440-43-9
8	^a^benzo(A)pyrene	1306	50-32-8
9	Polycyclic aromatic hydrocarbons	1277	130,498-29-2
10	Benzo(b)fluoranthene	1255	205-99-2

ATSDR, Agency for Toxic Substances and Disease Registry

a Metal-metalloid neurotoxicants.

## Data Availability

No data were used for the research described in the article.

## References

[R1] Andrews SJ, : The complex genetic architecture of Alzheimer’s disease: novel insights and future directions. EBioMedicine 2023, 90:104511, 10.1016/j.ebiom.2023.104511. PMC10024184 36907103

[2] Karlsson IK, : Measuring heritable contributions to Alzheimer’s disease: polygenic risk score analysis with twins. Brain Commun 2022, 4:fcab308, 10.1093/braincomms/fcab308. PMC8833403 35169705

[3] Migliore L, Coppede F: Gene-environment interactions in Alzheimer disease: the emerging role of epigenetics. Nat Rev Neurol 2022, 18:643–660, 10.1038/s41582-022-00714-w. 36180553

[4] Bakulski KM, : Heavy metals exposure and Alzheimer’s disease and related dementias. J Alzheimers Dis 2020, 76: 1215–1242, 10.3233/JAD-200282. 32651318 PMC7454042

[5] Ravichandran J, Karthikeyan BS, Singla P, Aparna SR, Samal A: NeurotoxKb 1.0: compilation, curation and exploration of a knowledgebase of environmental neurotoxicants specific to mammals. Chemosphere 2021, 278:130387, 10.1016/j.chemosphere.2021.130387. 33838427

[6] Breijyeh Z, Karaman R: Comprehensive review on Alzheimer’s disease: causes and treatment. Molecules 2020, 25, 10.3390/molecules25245789. 33302541 PMC7764106

[R7] ATSDR (Agency for Toxic Substances and Disease Registry): ATSDR’s substance priority list. 2024.

[8] Adkins-Jackson PB, : The structural and social determinants of Alzheimer’s disease related dementias. Alzheimer’s Dement 2023, 19:3171–3185, 10.1002/alz.13027. 37074203 PMC10599200

[9] Pierce BL, : Arsenic metabolism efficiency has a causal role in arsenic toxicity: mendelian randomization and gene-environment interaction. Int J Epidemiol 2013, 42:1862–1871, 10.1093/ije/dyt182. 24536095 PMC3887566

[10] Bustaffa E, Stoccoro A, Bianchi F, Migliore L: Genotoxic and epigenetic mechanisms in arsenic carcinogenicity. Arch Toxicol 2014, 88:1043–1067, 10.1007/s00204-014-1233-7. 24691704

[11] National Research Council: Critical aspects of EPA’s IRIS assessment of inorganic arsenic: interim report. 2013. Washington, DC.

[12] Straif K, : A review of human carcinogens–Part C: metals, arsenic, dusts, and fibres. Lancet Oncol 2009, 10:453–454, 10.1016/s1470-2045(09)70134-2. 19418618

[13] National Toxicology Program: Report on carcinogens, fifteenth edition: arsenic and inorganic arsenic compounds. Department of Health and Human Services; 2021.

[14] Environmental Protection Agency (EPA): National primary drinking water regulations; arsenic and clarifications to compliance and new source contaminants monitoring. Report no. WH-FRL-6934–9. Federal Register; 2001.

[15] World Health Organization (WHO): Guidelines for drinking-water quality. 4th ed. incorporating the 1st addendum; 2017:631. Report No. ISBN: 978-92-4-154995-0.

[16] Carmona A, Roudeau S, Ortega R: Molecular mechanisms of environmental metal neurotoxicity: a focus on the interactions of metals with synapse structure and function. Toxics 2021, 9, 10.3390/toxics9090198. PMC8471991 34564349

[17] Escudero-Lourdes C: Toxicity mechanisms of arsenic that are shared with neurodegenerative diseases and cognitive impairment: role of oxidative stress and inflammatory responses. Neurotoxicology 2016, 53:223–235, 10.1016/j.neuro.2016.02.002. 26868456

[18] Alboghobeish S, Pashmforosh M, Zeidooni L, Samimi A, Rezaei M: High fat diet deteriorates the memory impairment induced by arsenic in mice: a sub chronic in vivo study. Metab Brain Dis 2019, 34:1595–1606, 10.1007/s11011-019-00467-4. 31422513

[19] Wisessaowapak C, Visitnonthachai D, Watcharasit P, Satayavivad J: Prolonged arsenic exposure increases tau phosphorylation in differentiated SH-SY5Y cells: the contribution of GSK3 and ERK1/2. Environ Toxicol Pharmacol 2021, 84:103626, 10.1016/j.etap.2021.103626. 33621689

[20] Niño SA, : Arsenic exposure contributes to the bio-energetic damage in an Alzheimer’s disease model. ACS Chem Neurosci 2019, 10:323–336, 10.1021/acschemneuro.8b00278. 30141907

[21] Zarazúa S, Bürger S, Delgado JM, Jiménez-Capdeville ME, Schliebs R: Arsenic affects expression and processing of amyloid precursor protein (APP) in primary neuronal cells overexpressing the Swedish mutation of human APP. Int J Dev Neurosci : the official journal of the International Society for Developmental Neuroscience 2011, 29:389–396, 10.1016/j.ijdevneu.2011.03.004. 21440049

[22] Gong G, : Low-level groundwater arsenic exposure impacts cognition: a project FRONTIER study. J Environ Health 2011, 74:16–22. 21949980

[23] O’Bryant SE, Edwards M, Menon CV, Gong G, Barber R: Long-term low-level arsenic exposure is associated with poorer neuropsychological functioning: a project FRONTIER study. Int J Environ Res Publ Health 2011, 8:861–874, 10.3390/ijerph8030861. PMC3083674 21556183

[24] Baum L, : Serum zinc is decreased in Alzheimer’s disease and serum arsenic correlates positively with cognitive ability. Biometals 2010, 23:173–179, 10.1007/s10534-009-9277-5. 19911117

[25] Gong G, O’Bryant SE: The arsenic exposure hypothesis for Alzheimer disease. Alzheimer Dis Assoc Disord 2010, 24: 311–316, 10.1097/WAD.0b013e3181d71bc7. 20473132

[26] Dodson M, : Low-level arsenic causes proteotoxic stress and not oxidative stress. Toxicol Appl Pharmacol 2018, 341: 106–113, 10.1016/j.taap.2018.01.014. 29408041 PMC5929483

[27] Dodson M, : Increased O-GlcNAcylation of SNAP29 drives arsenic-induced autophagic dysfunction. Mol Cell Biol 2018, 38, 10.1128/mcb.00595-17. PMC5954189 29507186

[28] Wadgaonkar P, Chen F: Connections between endoplasmic reticulum stress-associated unfolded protein response, mitochondria, and autophagy in arsenic-induced carcino-genesis. Semin Cancer Biol 2021, 76:258–266, 10.1016/j.semcancer.2021.04.004. 33836253 PMC8492764

[29] Andersson S, : Genome-wide imaging screen uncovers molecular determinants of arsenite-induced protein aggregation and toxicity. J Cell Sci 2021, 134, 10.1242/jcs.258338. PMC8214759 34085697

[30] Fu SC, : Arsenic induces autophagy-dependent apoptosis via Akt inactivation and AMPK activation signaling pathways leading to neuronal cell death. Neurotoxicology 2021, 85:133–144, 10.1016/j.neuro.2021.05.008. 34038756

[31] Rahman MA, : Exposure to environmental arsenic and emerging risk of Alzheimer’s disease: perspective mechanisms, management strategy, and future directions. Toxics 2021, 9, 10.3390/toxics9080188. PMC8402411 34437506

[32] World Health Organization (WHO): Exposure to lead: a major public health concern. 2023.

[33] World Health Organization (WHO): Inorganic lead: Report no. Environmental health criteria 165. International Programme on Chemical Safety; 1995.

[34] Environmental Protection Agency (EPA): Integrated science assessment (ISA) for lead. Report no. EPA/600/R-10/075F. 2013.

[35] Lanphear BP, Rauch S, Auinger P, Allen RW, Hornung RW: Low-level lead exposure and mortality in US adults: a population-based cohort study. Lancet Public Health 2018, 3:e177–e184, 10.1016/S2468-2667(18)30025-2. 29544878

[36] ATSDR (Agency for Toxic Substances and Disease Registry): What are U.S. standards for lead levels? Report no. Course: WB2832. 2017.

[37] Weisskopf MG, : Association of cumulative lead exposure with Parkinson’s disease. Environ Health Perspect 2010, 118:1609–1613, 10.1289/ehp.1002339. 20807691 PMC2974701

[38] Choi YH, Park SK: Environmental exposures to lead, mercury, and cadmium and hearing loss in adults and adolescents: KNHANES 2010–2012. Environ Health Perspect 2017, 125, 067003, 10.1289/ehp565. 28599263 PMC5743444

[39] Bakulski KM, Rozek LS, Dolinoy DC, Paulson HL, Hu H: Alzheimer’s disease and environmental exposure to lead: the epidemiologic evidence and potential role of epigenetics. Curr Alzheimer Res 2012, 9:563–573, 10.2174/156720512800617991. 22272628 PMC3567843

[40] Farooqui Z, : Associations of cumulative Pb exposure and longitudinal changes in Mini-mental status exam scores, global cognition and domains of cognition: the VA normative aging study. Environ Res 2017, 152:102–108, 10.1016/j.envres.2016.10.007. 27770710 PMC5135609

[41] Payton M, Riggs KM, Spiro A 3rd, Weiss ST, Hu H: Relations of bone and blood lead to cognitive function: the VA normative aging study. Neurotoxicol Teratol 1998, 20:19–27, 10.1016/s0892-0362(97)00075-5. 9511166

[42] Shih RA, : Environmental lead exposure and cognitive function in community-dwelling older adults. Neurology 2006, 67: 1556–1562, 10.1212/01.wnl.0000239836.26142.c5. 16971698

[43] Gerhardsson L, Lundh T, Londos E, Minthon L: Cerebrospinal fluid/plasma quotients of essential and non-essential metals in patients with alzheimer’s disease. J Neural Transm 2011, 118:957–962, 10.1007/s00702-011-0605-x. 21373763

[44] Ramirez Ortega D, : Cognitive impairment induced by lead exposure during lifespan: mechanisms of lead neurotoxicity. Toxics 2021, 9, 10.3390/toxics9020023. PMC7912619 33525464

[45] Rocha A, Trujillo KA: Neurotoxicity of low-level lead exposure: history, mechanisms of action, and behavioral effects in humans and preclinical models. Neurotoxicology 2019, 73: 58–80, 10.1016/j.neuro.2019.02.021. 30836127 PMC7462347

[46] Toscano CD, Guilarte TR: Lead neurotoxicity: from exposure to molecular effects. Brain Res Brain Res Rev 2005, 49: 529–554, 10.1016/j.brainresrev.2005.02.004. 16269318

[47] Rice D, Barone S Jr: Critical periods of vulnerability for the developing nervous system: evidence from humans and animal models. Environ Health Perspect 2000, 108(Suppl 3): 511–533, 10.1289/ehp.00108s3511. 10852851 PMC1637807

[48] Masoud AM, Bihaqi SW, Machan JT, Zawia NH, Renehan WE: Early-life exposure to lead (Pb) alters the expression of microRNA that target proteins associated with Alzheimer’s disease. J Alzheimers Dis 2016, 51:1257–1264, 10.3233/jad-151018. 26923026

[49] Bihaqi SW, Bahmani A, Adem A, Zawia NH: Infantile post-natal exposure to lead (Pb) enhances tau expression in the cerebral cortex of aged mice: relevance to AD. Neurotoxicology 2014, 44:114–120, 10.1016/j.neuro.2014.06.008. 24954411 PMC4175119

[50] Behl M, Zhang Y, Monnot AD, Jiang W, Zheng W: Increased beta-amyloid levels in the choroid plexus following lead exposure and the involvement of low-density lipoprotein receptor protein-1. Toxicol Appl Pharmacol 2009, 240: 245–254, 10.1016/j.taap.2009.05.024. 19501112 PMC2753690

[51] Gassowska M, : Perinatal exposure to lead (Pb) promotes tau phosphorylation in the rat brain in a GSK-3beta and CDK5 dependent manner: relevance to neurological disorders. Toxicology 2016, 347–349:17–28, 10.1016/j.tox.2016.03.002. 27012722

[52] Zhang XL, : Presynaptic mechanisms of lead neurotoxicity: effects on vesicular release, vesicle clustering and mitochondria number. PLoS One 2015, 10, e0127461, 10.1371/journal.pone.0127461. PMC4444102 26011056

[53] Li Y, : Biometal dyshomeostasis and toxic metal accumulations in the development of Alzheimer’s disease. Front Mol Neurosci 2017, 10:339, 10.3389/fnmol.2017.00339. 29114205 PMC5660707

[R54] Gu H, : Lead acetate exposure and cerebral amyloid accumulation: mechanistic evaluations in APP/PS1 mice. Environ Health Perspect 2024, 132:107004, 10.1289/EHP14384. PMC11482597 39412896

[55] Gu H, : Evaluation of chronic lead effects in the blood brain barrier system by DCE-CT. J Trace Elem Med Biol 2020, 62:126648, 10.1016/j.jtemb.2020.126648. PMC7655551 32980769

[56] Li H, : Associations between blood cadmium levels and cognitive function in a cross-sectional study of US adults aged 60 years or older. BMJ Open 2018, 8, e020533, 10.1136/bmjopen-2017-020533. PMC5898350 29654035

[57] Ciesielski T, Bellinger DC, Schwartz J, Hauser R, Wright RO: Associations between cadmium exposure and neuro-cognitive test scores in a cross-sectional study of US adults. Environ Health : a global access science source 2013, 12:13, 10.1186/1476-069x-12-13. PMC3599125 23379984

[58] Min JY, Min KB: Blood cadmium levels and Alzheimer’s disease mortality risk in older US adults. Environ Health : a global access science source 2016, 15:69, 10.1186/s12940-016-0155-7. PMC4908725 27301955

[59] Peng Q, Bakulski KM, Nan B, Park SK: Cadmium and Alzheimer’s disease mortality in U.S. adults: updated evidence with a urinary biomarker and extended follow-up time. Environ Res 2017, 157:44–51, 10.1016/j.envres.2017.05.011. 28511080 PMC5513740

[60] World Health Organization (WHO): Cadmium. Report no. Environmental health criteria 134. (International Programme on Chemical Safety (Environmental Health Criteria 134); 1992.

[61] Notarachille G, Arnesano F, Calò V, Meleleo D: Heavy metals toxicity: effect of cadmium ions on amyloid beta protein 1–42. Possible implications for Alzheimer’s disease. Biometals 2014, 27:371–388, 10.1007/s10534-014-9719-6. 24557150

[62] Li X, Lv Y, Yu S, Zhao H, Yao L: The effect of cadmium on Abeta levels in APP/PS1 transgenic mice. Exp Ther Med 2012, 4:125–130, 10.3892/etm.2012.562. 23060935 PMC3460286

[63] Tobwala S, Wang H-J, Carey JW, Banks WA, Ercal N: Effects of lead and cadmium on brain endothelial cell survival, monolayer permeability, and crucial oxidative stress markers in an in vitro model of the blood-brain barrier. Toxics 2014, 2:258–275.

[64] Ashok A, Rai NK, Tripathi S, Bandyopadhyay S: Exposure to As-, Cd-, and Pb-mixture induces Aβ, amyloidogenic APP processing and cognitive impairments via oxidative stress-dependent neuroinflammation in young rats. Toxicol Sci : an official journal of the Society of Toxicology 2015, 143:64–80, 10.1093/toxsci/kfu208. 25288670

[65] Xu B, : Calcium signaling is involved in cadmium-induced neuronal apoptosis via induction of reactive oxygen species and activation of MAPK/mTOR network. PLoS One 2011, 6, e19052, 10.1371/journal.pone.0019052. PMC3081326 21544200

[R66] Oblak AL, : Plcg2(M28L) interacts with high fat/high sugar diet to accelerate Alzheimer’s disease-relevant phenotypes in mice. Front Aging Neurosci 2022, 14, 886575, 10.3389/fnagi.2022.886575. PMC9263289 35813947

[67] Dunn AR, O’Connell KMS, Kaczorowski CC: Gene-by-environment interactions in Alzheimer’s disease and Parkinson’s disease. Neurosci Biobehav Rev 2019, 103:73–80, 10.1016/j.neubiorev.2019.06.018. 31207254 PMC6700747

[68] Islam F, : Exposure of metal toxicity in Alzheimer’s disease: an extensive review. Front Pharmacol 2022, 13, 903099, 10.3389/fphar.2022.903099. PMC9465172 36105221

[69] Xie J, : Developmental Pb exposure increases AD risk via altered intracellular Ca(2+) homeostasis in hiPSC-derived cortical neurons. J Biol Chem 2023, 299:105023, 10.1016/j.jbc.2023.105023. PMC10413359 37423307

[70] Kotredes KP, : Uncovering disease mechanisms in a novel mouse model expressing humanized APOEepsilon4 and Trem2*R47H. Front Aging Neurosci 2021, 13, 735524, 10.3389/fnagi.2021.735524. PMC8544520 34707490

[R71] Kotredes KP, : Characterizing molecular and synaptic signatures in mouse models of late-onset Alzheimer’s disease independent of amyloid and tau pathology. Alzheimer’s Dement 2024, 20:4126–4146, 10.1002/alz.13828. 38735056 PMC11180851

[R72] Preuss C, : A novel systems biology approach to evaluate mouse models of late-onset Alzheimer’s disease. Mol Neurodegener 2020, 15:67, 10.1186/s13024-020-00412-5. 33172468 PMC7656729

[73] Wan YW, : Meta-analysis of the Alzheimer’s disease human brain transcriptome and functional dissection in mouse models. Cell Rep 2020, 32:107908, 10.1016/j.celrep.2020.107908. PMC7428328 32668255

[R74] Baccarelli A, Dolinoy DC, Walker CL: A precision environmental health approach to prevention of human disease. Nat Commun 2023, 14:2449, 10.1038/s41467-023-37626-2. 37117186 PMC10147599

[75] Bollati V, Baccarelli A: Environmental epigenetics. Heredity 2010, 105:105–112, 10.1038/hdy.2010.2. 20179736 PMC3133724

[R76] Wang T, : The NIEHS TaRGET II consortium and environmental epigenomics. Nat Biotechnol 2018, 36:225–227, 10.1038/nbt.4099. 29509741 PMC5991835

[77] Barouki R, : Epigenetics as a mechanism linking developmental exposures to long-term toxicity. Environ Int 2018, 114:77–86, 10.1016/j.envint.2018.02.014. 29499450 PMC5899930

[78] Calderon-Garciduenas L, : Reduced repressive epigenetic marks, increased DNA damage and Alzheimer’s disease hallmarks in the brain of humans and mice exposed to particulate urban air pollution. Environ Res 2020, 183:109226, 10.1016/j.envres.2020.109226. 32045727

[79] Kovatsi L, : p16 promoter methylation in Pb2+ -exposed individuals. Clin Toxicol 2010, 48:124–128, 10.3109/15563650903567091. 20199129

[80] O’Connell KMS, Ouellette AR, Neuner SM, Dunn AR, Kaczorowski CC: Genetic background modifies CNS-mediated sensorimotor decline in the AD-BXD mouse model of genetic diversity in Alzheimer’s disease. Gene Brain Behav 2019, 18, e12603, 10.1111/gbb.12603. PMC6899779 31381246

